# Secondary prevention of coronary heart disease: a cross-sectional analysis on the Brazilian Longitudinal Study of Adult Health (ELSA-Brasil)

**DOI:** 10.1590/1516-3180.2018.0531140319

**Published:** 2019-08-29

**Authors:** Marina Gabriela Birck, Alessandra Carvalho Goulart, Paulo Andrade Lotufo, Isabela Martins Benseñor

**Affiliations:** I BSc, MSc. Postgraduate Student, Faculdade de Medicina da Universidade de São Paulo (FMUSP), São Paulo (SP), Brazil.; II MD, PhD. Clinical Epidemiologist and Researcher, Center for Clinical and Epidemiological Research, Hospital Universitário da Universidade de São Paulo (HU-USP), São Paulo (SP), Brazil.; III MD, DrPH. Full Professor, Department of Internal Medicine, Faculdade de Medicina da Universidade de São Paulo (FMUSP), São Paulo (SP), Brazil.; IV MD, PhD. Professor, Department of Internal Medicine, and Director of Center for Clinical and Epidemiological Research, Hospital Universitário da Universidade de São Paulo (HU-USP), São Paulo (SP), Brazil.

**Keywords:** Heart diseases, Coronary disease, Secondary prevention, Risk factors, Cohort studies

## Abstract

**BACKGROUND:**

Coronary heart disease (CHD) remains a major cause of mortality worldwide and in Brazil. Use of standard medications after CHD has been proven to avoid new events and reduce early mortality.

**OBJECTIVES:**

This study aimed to analyze secondary prevention of CHD and its association with the baseline characteristics of the Brazilian Longitudinal Study of Adult Health (ELSA-Brasil).

**DESIGN AND SETTING:**

Cross-sectional analysis on ELSA-Brasil data.

**METHODS:**

Secondary prevention of CHD recommended in standard guidelines (antiplatelet plus beta-blocker plus lipid-lowering drug, with or without angiotensin-converting enzyme inhibitors, ACEI, or angiotensin receptor blockers, ARB) was evaluated in relation to sociodemographic data and the time since the coronary event. The chi-square test, one-way analysis of variance (ANOVA) and Mann-Whitney test were performed, as necessary.

**RESULTS:**

Among 15,094 participants, 2.7% reported a previous diagnosis of CHD. Use of recommended drugs for secondary prevention was reported by almost 35% of the participants. Medication use for secondary prevention was generally more frequent among high-income participants than among low-income participants. Use of ARB and ACEI was different between participants who had private health insurance and those who only used the public healthcare system. Men were more likely to use medication than women. The frequency with which participants used the recommended drugs was similar in all time periods after CHD, but use of only one drug increased progressively across time periods.

**CONCLUSION:**

The use of medication for secondary prevention of CHD was lower than what is recommended in standardized guidelines, especially among women and lower-income participants.

## INTRODUCTION

Coronary heart disease (CHD) remains a major cause of mortality worldwide and in Brazil. As life expectancy has increased in low-middle income countries, including Brazil, and people start to live long enough to develop CHD, there has been an increase in the years of life lost (YLL) that possibly presents a correlation with suboptimal access to healthcare in these areas.^1^ In Brazil, late case-fatality in occurrences of CHD is a very important problem that has been found to be associated with poor follow-up after an acute coronary event.^2^

In this context, secondary prevention of CHD, especially using pharmacological therapy, has been effective in reducing recurrence of events, decreasing morbidity and mortality and improving quality of life.^3^ National and international guidelines recommend long-term use of evidence-based medication for management of CHD, such as use of acetylsalicylic acid (ASA), beta blockers, angiotensin-converting enzyme inhibitors (ACEI) and statins, as first-line therapy; or other antiplatelet medication, angiotensin receptor blocker (ARB) or fibrate when first-line therapy is contraindicated.^3^^,^^4^^,^^5^^,^^6^^,^^7^^,^^8^ Despite this well-established knowledge, not all patients with CHD are able to obtain the standard treatment endorsed by the guidelines. Lack of adherence to these medications is associated with higher risk of adverse outcomes. Furthermore, sociodemographic factors are known to influence medication use and prescription, and all these factors need to be taken into consideration when developing interventions and public policies that are aimed towards improving adherence.^9^^,^^10^^,^^11^^,^^12^

## OBJECTIVE

We sought to analyze secondary prevention of CHD and its association with the baseline characteristics of the Brazilian Longitudinal Study of Adult Health (ELSA-Brasil).

## METHODS

### Participants

ELSA-Brasil is a cohort study on 15,105 civil servants aged 35 to 74 years living in six cities (Salvador, Vitória, Belo Horizonte, Rio de Janeiro, São Paulo and Porto Alegre). It was designed to investigate cardiovascular diseases and diabetes, and their associated factors.^13^ The exclusion criteria were current or recent pregnancy (< 4 months prior to the first interview), intention to leave employment at the institution in the near future, severe cognitive or communication impairment or living outside of the study center’s corresponding metropolitan area. The baseline assessment was made between August 2008 and December 2010 and included application of validated questionnaires and clinical and laboratory examinations.^13^^,^^14^^,^^15^^,^^16^

All the participants in the study were invited to visit the research center to answer a detailed questionnaire on their sociodemographic and clinical risk factors, along with their medication use. The training for all interviewers was centralized, with certification and recertification every six months for all interviewers and for all the people who performed measurements and laboratory tests. Also, during data collection, periodic staff meetings were held to discuss problems and to check whether standardized procedures were correctly performed.^13^

The study protocol followed the ethical guidelines of the 1975 Declaration of Helsinki. Approvals were granted by the institutional review boards of all centers [Ethics Committees of Hospital de Clínicas de Porto Alegre (under the registration number 194/06), Hospital Universitário da Universidade de São Paulo (669/06), Fundação Oswaldo Cruz (343/06), Universidade Federal de Minas Gerais (186/06), Universidade Federal da Bahia (027/06) and Universidade Federal do Espírito Santo (041/06)] and all participants provided written informed consent.^17^

In this cross-sectional baseline analysis, we included all participants for whom self-reported information about CHD was available (N = 15,094).

### Variables

#### Coronary heart disease

CHD was considered to be present if it was self-reported at the baseline assessment, through reports of a medical history of myocardial infarction and/or percutaneous coronary intervention, including balloon angioplasty with or without stent placement, or myocardial revascularization.

#### Medication use

All participants were asked about their continuous and non-continuous use of prescription and nonprescription medication over the previous two weeks. They were instructed to bring all medications to the study center. Seven different categories were created for medication use: ASA, any beta blocker, ACEI, statins, other antiplatelet medications, ARB and fibrates. A description of the drugs included is provided in Supplemental File 1.

#### Time since CHD

The time that had elapsed since the occurrence of CHD was defined as the difference between the date of the interview and the date of the event, as reported by the participants. When both infarction and coronary intervention were reported, the oldest event was used. This variable was divided into the following categories: ≤ 4, 5-9, 10-14 and ≥ 15 years.

#### Covariates

This study considered the following sociodemographic variables: age (years), sex, self-declared race (white, mixed, black, Asian or indigenous), education (less than high school, completed high school and some college/university, or completed college/university or more), mean monthly family income (≤ USD 1245, USD 1246-3319 or ≥ USD 3320) and whether the participants had private health insurance. Local currency (Brazilian reais, BRL) was converted to U.S. dollars (USD) at the prevailing rate in December 2008, of BRL 2.00 = USD 1.00.

Anthropometric measurements were obtained using standard protocols.^18^ Body mass index (BMI) was calculated as weight (in kilograms) divided by height squared (in meters).

A family history of premature cardiovascular disease was taken to mean a diagnosis of CHD, including myocardial infarction, revascularization or sudden death in a first-degree relative before the age of 60 years. Smoking and alcohol use were categorized as never, past or current. Presence of hypertension was defined as use of antihypertensive medication, or systolic blood pressure ≥ 140 mmHg, or diastolic blood pressure ≥ 90 mmHg.

Presence of diabetes mellitus was defined as a previous medical diagnosis, use of medication to treat diabetes, fasting plasma glucose ≥ 7.0 mmol/l (≥ 126 mg/dl), two-hour plasma glucose after an oral glucose overload as part of an oral glucose tolerance test ≥ 11.1 mmol/l (≥ 200 mg/dl) or glycated hemoglobin (HbA1c) ≥ 6.5% (≥ 47.5 mmol/mol). Presence of dyslipidemia was defined as an LDL-cholesterol level ≥ 130 mg/dl or use of lipid-lowering medication. Presence of chronic kidney disease (CKD) was defined as a glomerular filtration rate (calculated by means of CKDEpi) < 60 ml/min/1.73 m^2^. The strategies used for collection, processing and quality control of the blood and urine tests in ELSA-Brasil have been published previously.^19^

Depression was assessed through the Clinical Interview Schedule-Revised (CIS-R), which is a structured interview used to diagnose current common nonpsychotic conditions.^20^^,^^21^

Dietary quality was assessed using the Brazilian Healthy Eating Index Revised (BHEI-R), via a score ranging from 0 to 100.^22^ Physical activity during leisure time and commuting was assessed through the International Physical Activity Questionnaire (IPAQ) long form,^23^^,^^24^^,^^25^ and the participants were categorized as active, insufficiently active or inactive, as recommended by the World Health Organization (WHO).

Adherence to medication was assessed using the four-item Morisky Medication Adherence Scale (MMAS-4). This method consisted of asking four questions about the use of continuous medication: (1) Sometimes if you feel worse when you take the medicine, do you stop taking it? (2) When you feel better, do you sometimes stop taking your medicine? (3) Are you careless at times about taking your medicine? and (4) Do you ever forget to take your medicine? The participants were considered to be adherent if they answered “no” to all questions. Nonadherence consisted of at least one positive answer.^26^

### Statistical analysis

Categorical variables were compared using the chi-square test, and were presented as absolute numbers and proportions. Continuous variables were tested for normality using the Kolmogorov-Smirnov goodness-of-fit test. They were compared either using one-way analysis of variance (ANOVA), with presentation as means and standard deviations; or using the Mann-Whitney test, with presentation as medians and interquartile ranges, as appropriate. In addition, associations of sociodemographic variables and medication use (antiplatelet drug plus beta blocker plus lipid-lowering drug, with or without ACEI or ARB) were assessed by means of logistic regression models. Crude models (univariate analysis) and an adjusted model (taking into account income, education, sex, race, age and private health insurance) were built and presented with the odds ratio (OR) and 95% confidence interval (95% CI).

All the analyses were performed using the Statistical Package for the Social Sciences software, version 22 (SPSS Inc., Chicago, Illinois, USA). P-values < 0.05 were considered statistically significant.

## RESULTS

Among the 15,094 participants, 405 (2.7%) reported having a prior history of CHD. The frequency of CHD was higher among men and participants in the lower socioeconomic level (less education and lower mean family monthly income). The participants with CHD had higher BMI and, especially, larger waist circumference, as well as higher frequencies of hypertension, diabetes, dyslipidemia, CKD and depression. They presented lower HDL-cholesterol and LDL-cholesterol levels than those of non-CHD participants. In the case of LDL-c, the lower levels may be explained by reverse causation, with prescription of statins for patients with CHD. The participants with CHD presented slightly higher quality in their diets, but although this was statistically significant, the differences were not clinically relevant. They were less active during leisure time, but no difference was found in relation to commuting. The participants with CHD had higher frequency of adhering to continuous medication than did those without the disease (Table 1).

**Table 1.** Characteristics of participants with self-reported coronary heart disease at the baseline of the Brazilian Longitudinal Study of Adult Health (ELSA-Brasil)

	Coronary heart disease		P-value
	No n = 14,689 (%)	Yes n = 405 (%)	
Age (years)*	51.8 (9.0)	60.9 (8.1)	< 0.0001
Age stratum (%)			
35-44	3,333 (22.7)	7 (1.7)	< 0.0001
45-54	5,848 (39.8)	86 (21.2)	
55-64	4,060 (27.6)	172 (42.5)	
65-74	1,448 (9.9)	140 (34.6)	
Female (%)	8,065 (54.9)	148 (36.5)	< 0.0001
Race (%)			
White	7,574 (52.2)	212 (53.5)	0.199
Mixed	4,102 (28.3)	98 (24.7)	
Black	2,325 (16.0)	69 (17.4)	
Asian	364 (2.5)	9 (2.3)	
Indigenous	149 (1.0)	8 (2.0)	
Educational level (%)			
Less than high school	1,803 (12.3)	114 (28.1)	< 0.0001
High school and some college/university	4,108 (28.0)	94 (23.2)	
Completed college/university or more	8,778 (59.8)	197 (48.6)	
Mean monthly family income (%)			
≤ USD 1,245	3,868 (26.4)	121 (30.1)	0.026
USD 1,246 to 3,319	5,579 (38.1)	127 (31.6)	
≥ USD 3,320	5,178 (35.4)	154 (38.3)	
Private health insurance (%)	10,024 (68.2)	272 (67.2)	0.643
Smoker (%)			
Never	8,423 (57.3)	164 (40.5)	< 0.0001
Past	4,332 (29.5)	197 (48.6)	
Current	1,933 (13.2)	44 (10.9)	
Alcohol consumption (%)			
Never	1,545 (10.5)	66 (16.5)	< 0.0001
Past	2,927 (20.0)	104 (25.9)	
Current	10,195 (69.5)	231 (57.6)	
Dietary quality score*	69.7 (9.3)	71.3 (10.9)	0.004
Leisure-time physical activity (%)			
Inactive	9,129 (63.1)	286 (71.3)	0.003
Insufficiently active	1,830 (12.6)	42 (10.5)	
Active	3,517 (24.3)	73 (18.2)	
Commuting physical activity (%)			
Inactive	3,856 (26.7)	106 (26.4)	0.884
Insufficiently active	5,608 (38.8)	152 (37.9)	
Active	4,984 (34.5)	143 (35.7)	
Family history (%)	3,052 (21.1)	139 (35.9)	< 0.0001
Dyslipidemia (%)	8,331 (57.2)	326 (81.3)	< 0.0001
Hypertension (%)	5,090 (34.7)	304 (75.2)	< 0.0001
Diabetes mellitus (%)	2,796 (19.0)	171 (42.2)	< 0.0001
Chronic kidney disease (%)	902 (6.2)	75 (18.7)	< 0.0001
Depression (%)	607 (4.1)	29 (7.2)	0.003
Body mass index (kg/m²)*	27.0 (4.8)	28.1 (4.6)	< 0.0001
Waist circumference (cm)*	91.1 (12.9)	97.0 (12.2)	< 0.0001
Triglycerides (mg/dl)**	114.0 (81;165)	127.0 (95;173)	< 0.0001
LDL cholesterol (mg/dl)*	131.5 (34.8)	114.1 (40.5)	< 0.0001
HDL cholesterol (mg/dl)*	56.8 (14.6)	51.54 (13.1)	< 0.0001
Medication adherence (%)			
Adherent	3,130 (37.0)	163 (44.3)	0.005
Non-adherent	5,321 (63.0)	205 (55.7)	

USD = United States dollars; LDL = low-density lipoprotein; HDL = high-density lipoprotein.

*Presented as the mean (with SD); **Presented as the median (with interquartile interval).

Tables 2**and**3 show the associations of medication use and sociodemographic characteristics among the participants who self-reported CHD (first and second-line therapy for secondary prevention, respectively). Older participants reported having higher frequencies of use of ASA, statins, other antiplatelet medications and ARB, but no differences were found in relation to beta blockers and ACEI. Except for ARB, medication use for secondary prevention of CHD was higher among men than among women. Use of ASA, other antiplatelet medications and statins was higher among whites and those with higher socioeconomic status (higher education and income), except for beta blockers, for which use was higher among individuals with high income but not among whites or individuals with higher education levels. Having private health insurance was associated with use of ASA, other antiplatelet medications, ARB and statins, and with no use of ACEI. No association was found between fibrate use and any sociodemographic characteristic. Adhering to continuous treatment was associated with use of all first-line medications, except ACEI.

**Table 2.** Sociodemographic variables associated with use of first-line therapy among participants with self-reported coronary heart disease

	ASA			Beta blockers			ACEI			Statins		
	No n = 184	Yes n = 220	P-value	No n = 189	Yes n = 215	P-value	No n = 263	Yes n = 141	P-value	No n = 163	Yes n = 241	P-value
Age (mean, SD)	59.3 (8.3)	62.2 (7.6)	0.0004	60.2 (8.4)	61.4 (7.7)	0.145	60.4 (8.4)	61.8 (7.3)	0.082	58.7 (8.5)	62.3 (7.4)	< 0.0001
Age stratum (%)												
35-44	7 (3.8)	0 (0.0)	0.002	6 (3.2)	1 (0.2)	0.148	7 (2.7)	0 (0.0)	0.111	7 (4.3)	0 (0.0)	< 0.0001
45-54	47 (25.5)	39 (17.7)		43 (22.8)	43 (20.0)		61 (23.2)	25 (17.7)		45 (27.6)	41 (17.0)	
55-64	78 (42.4)	94 (42.7)		80 (42.3)	92 (42.8)		106 (40.3)	66 (46.8)		71 (43.6)	101 (41.9)	
65-74	52 (28.3)	87 (39.5)		60 (31.7)	79 (19.6)		89 (33.8)	50 (35.5)		40 (24.5)	99 (41.1)	
Sex (%)												
Male	96 (52.2)	160 (72.7)	< 0.0001	109 (57.7)	147 (68.4)	0.026	156 (59.3)	100 (70.9)	0.021	78 (47.9)	178 (73.9)	< 0.0001
Female	88 (47.8)	60 (27.3)		80 (42.3)	68 (31.6)		107 (40.7)	41 (29.1)		85 (52.1)	63 (26.1)	
Race (%)												
White	79 (43.4)	133 (62.1)	0.0003	88 (46.8)	124 (59.6)	0.076	133 (52.0)	79 (56.4)	0.706	64 (39.8)	148 (63.0)	< 0.0001
Mixed	48 (26.4)	50 (23.4)		50 (26.6)	48 (23.1)		68 (26.6)	30 (21.4)		45 (28.0)	53 (22.6)	
Black	47 (25.8)	22 (10.3)		39 (20.7)	30 (14.4)		43 (16.8)	26 (18.6)		43 (26.7)	26 (11.1)	
Asian	5 (2.7)	4 (1.9)		5 (2.7)	4 (1.9)		7 (2.7)	2 (1.4)		4 (2.5)	5 (2.1)	
Indigenous	3 (1.6)	5 (2.3)		6 (3.2)	2 (1.0)		5 (2.0)	3 (2.1)		5 (3.1)	3 (1.3)	
Education (%)												
Less than high school	59 (32.1)	55 (25.0)	0.009	61 (32.3)	53 (24.7)	0.062	67 (25.5)	47 (33.3)	0.229	55 (33.7)	59 (24.5)	< 0.0001
High school and some college/university	51 (27.7)	43 (19.5)		48 (25.4)	46 (21.4)		65 (24.7)	29 (20.6)		57 (35.0)	37 (15.4)	
Completed college/university or more	74 (40.2)	122 (55.5)		80 (42.3)	116 (54.0)		131 (49.8)	65 (46.1)		51 (31.3)	145 (60.2)	
Income (%)												
≤ USD 1,245	66 (36.3)	55 (25.0)	< 0.0001	64 (34.0)	57 (26.6)	0.008	78 (29.9)	43 (30.5)	0.705	71 (43.8)	50 (20.8)	< 0.0001
USD 1,246 to 3,319	68 (37.4)	59 (26.8)		67 (35.6)	60 (28.0)		86 (33.0)	41 (29.1)		59 (36.4)	68 (28.3)	
≥ USD 3,320	48 (26.4)	106 (48.2)		57 (30.3)	97 (45.3)		97 (37.2)	57 (40.4)		32 (19.8)	122 (50.8)	
Private health insurance (%)	114 (62.0)	157 (71.4)	0.045	128 (67.7)	143 (66.5)	0.796	189 (71.9)	82 (58.2)	0.005	92 (56.4)	179 (74.3)	< 0.0001
Medication adherence (%)												
Adherent	49 (32.7)	114 (52.3)	0.0002	57 (37.0)	106 (49.5)	0.017	95 (41.9)	68 (48.2)	0.231	35 (27.1)	128 (53.6)	< 0.0001
Non-adherent	101 (67.3)	104 (47.7)		97 (63.0)	108 (50.5)		132 (58.1)	73 (51.8)		94 (72.9)	111 (46.4)	

ASA = acetylsalicylic acid; ACEI = angiotensin-converting enzyme inhibitor; SD = standard deviation; USD = United States dollars.

**Table 3.** Sociodemographic variables associated with use of second-line therapy among participants with self-reported coronary heart disease

	Other antiplatelet			ARB			Fibrate		
	No n = 349 (%)	Yes n = 55 (%)	P-value	No n = 332 (%)	Yes n = 72 (%)	P-value	No n = 398 (%)	Yes n = 6 (%)	P-value
Age (mean, SD)	60.3 (8.0)	64.4 (7.5)	< 0.0001	60.1 (8.0)	64.3 (7.4)	< 0.0001	60.9 (8.1)	60.3 (5.6)	0.873
Age stratum (%)									
35-44	7 (2.0)	0 (0.0)	0.001	7 (1.7)	0 (0.0)	0.001	7 (1.8)	0 (0.0)	0.670
45-54	78 (22.3)	8 (14.5)		77 (23.2)	9 (12.5)		85 (21.4)	1 (16.7)	
55-64	157 (45.0)	15 (27.3)		148 (44.6)	24 (33.3)		168 (42.2)	4 (66.7)	
65-74	107 (30.7)	32 (58.2)		100 (30.1)	39 (54.2)		138 (34.7)	1 (16.7)	
Sex (%)									
Male	214 (61.3)	42 (76.4)	0.031	215 (64.8)	41 (56.9)	0.212	252 (63.3)	4 (66.7)	0.866
Female	135 (38.7)	13 (23.6)		117 (35.2)	31 (43.1)		146 (36.7)	2 (33.3)	
Race (%)									
White	173 (50.4)	39 (73.6)	0.034	179 (54.7)	33 (47.8)	0.319	208 (53.2)	4 (40.0)	0.771
Mixed	90 (26.2)	8 (15.1)		74 (22.6)	24 (34.8)		97 (24.8)	1 (20.0)	
Black	64 (18.7)	5 (9.4)		59 (18.0)	10 (14.5)		69 (17.6)	0 (0.0)	
Asian	8 (2.3)	1 (1.9)		8 (2.4)	1 (1.4)		9 (2.3)	0 (0.0)	
Indigenous	8 (2.3)	0 (0.0)		7 (2.1)	1 (0.3)		8 (2.0)	0 (0.0)	
Education (%)									
Less than high school	112 (32.1)	2 (3.6)	< 0.0001	90 (27.1)	24 (33.3)	0.108	112 (28.1)	2 (33.3)	0.917
High school and some college/university	81 (23.2)	13 (23.6)		84 (25.3)	10 (13.9)		93 (23.4)	1 (16.7)	
Completed college/university or more	156 (44.7)	40 (72.7)		158 (47.6)	38 (52.8)		193 (48.5)	3 (50.0)	
Income (%)									
≤ USD 1,245	114 (32.8)	7 (13.0)	< 0.0001	102 (30.8)	19 (26.8)	0.712	119 (30.1)	2 (33.3)	0.716
USD 1,246 to 3,319	115 (33.0)	12 (22.2)		102 (30.8)	25 (35.2)		126 (31.8)	1 (16.7)	
≥ USD 3,320	119 (34.2)	35 (64.8)		127 (38.4)	27 (38.0)		151 (38.1)	3 (50.0)	
Private health insurance (%)	227 (65.0)	44 (80.0)	0.028	211 (63.6)	60 (83.3)	0.001	267 (67.1)	4 (66.7)	0.983
Medication adherence (%)									
Adherent	133 (42.4)	30 (55.6)	0.71	131 (44.3)	32 (44.4)	0.977	161 (44.5)	2 (33.3)	0.586
Non-adherent	181 (57.6)	24 (44.4)		165 (55.7)	40 (55.6)		201 (55.5)	4 (66.7)	

ARB = angiotensin receptor blockers; SD = standard deviation; USD = United States dollars.

After adjustment for sociodemographic variables through logistic regression, men and participants reporting higher income presented higher frequency of using the recommended drugs for secondary prevention of CHD (Supplemental File 2). We also analyzed differences in sociodemographic variables according to sex, in an attempt to explain the lower use of secondary prevention among women than among men. Women with CHD presented higher frequency of lower education and income than seen among men, and more frequently reported themselves as having black or mixed color, than as having white skin color (Supplemental File 3).

The proportion of the participants who were not using any of the recommended drugs was approximately 16% and this was similar among all the periods of time since the occurrence of CHD (≤ 4, 5-9, 10-14 and ≥ 15 years), although it was higher among those with four years or less and with 15 years or more since the event. Recommended combinations (antiplatelet medication plus beta blocker plus lipid-lowering drugs, with or without ACEI/ARB) were used by around 35% and this proportion was also similar among different time periods. However, the most common situation regarding use of secondary prevention was the use of only one drug, which was reported by 11.8% of the participants with four years or less since the event and 21.7% of those with 15 years or more since the event (Table 4).

**Table 4.** Frequency of medication use according to drug class and time that had elapsed since coronary heart disease, at baseline of the Brazilian Longitudinal Study of Adult Health (ELSA-Brasil)

Drug class and combination	Time since CHD (years) n = 398 (%)				Total
	≤ 4	5-9	10-14	≥ 15	
Only ASA/other antiplatelet	3 (1.8)	4 (3.5)	1 (1.5)	1 (2.2)	9 (2.3)
Only beta blocker	3 (1.8)	6 (5.3)	2 (2.9)	1 (2.2)	12 (3.0)
Only ACEI/ARB	7 (4.1)	3 (2.7)	8 (11.8)	6 (13.0)	24 (6.0)
Only statin/fibrate	7 (4.1)	5 (4.4)	1 (1.5)	2 (4.3)	15 (3.8)
ASA/other antiplatelet + beta blocker	2 (1.2)	1 (0.9)	3 (4.4)	1 (2.2)	7 (1.8)
ASA/other antiplatelet + ACEI/ARB	6 (3.5)	2 (1.8)	3 (4.4)	1 (2.2)	12 (3.0)
ASA/other antiplatelet + statin/fibrate	10 (5.8)	9 (8.0)	6 (8.8)	1 (2.2)	26 (6.5)
Beta blocker + ACEI/ARB	7 (4.1)	3 (2.7)	2 (2.9)	1 (2.2)	13 (3.3)
Beta blocker + statin/fibrate	4 (2.3)	2 (1.8)	2 (2.9)	1 (2.2)	9 (2.3)
ACEI/ARB + statin/fibrate	7 (4.1)	5 (4.4)	1 (1.5)	1 (2.2)	14 (3.5)
ASA/other antiplatelet + beta blocker + ACEI/ARB	6 (3.5)	4 (3.5)	4 (5.9)	3 (6.5)	17 (4.3)
ASA/other antiplatelet + beta blocker + statin/fibrate	20 (11.7)	16 (14.2)	8 (11.8)	5 (10.9)	49 (12.3)
ASA/other antiplatelet + ACEI/ARB + statin/fibrate	12 (7.0)	6 (5.3)	2 (2.9)	1 (2.2)	21 (5.3)
Beta blocker + ACEI/ARB + statin/fibrate	5 (2.9)	7 (6.2)	1 (1.5)	3 (6.5)	16 (4.0)
ASA/other antiplatelet + beta blocker + ACEI/ARB + statin/fibrate	42 (24.6)	26 (23.0)	14 (15.2)	10 (21.7)	92 (23.1)
None of the recommended drugs	30 (17.5)	14 (12.4)	10 (14.7)	8 (17.4)	62 (15.6)
Total	171 (100)	113 (100)	68 (100)	46 (100)	398 (100)

CHD = coronary heart disease; ASA = acetylsalicylic acid; ACEI = angiotensin-converting enzyme inhibitors; ARB = angiotensin receptor blockers.

## DISCUSSION

In the baseline assessment of ELSA-Brasil, 2.7% of the participants self-reported having a previous diagnosis of CHD, with higher frequencies among men and older participants. As expected, lower educational and income levels, and presence of cardiovascular risk factors, such as hypertension, diabetes, central obesity, prior family history of CHD and sedentarism, were more common among participants reporting previous CHD at the baseline.

However, even though the ELSA-Brasil sample presented higher educational and income levels than those of the general population in Brazil, the use of recommended treatment for secondary prevention of CHD (antiplatelet plus beta blockers plus lipid-lowering drugs, with or without ACEI/ARB) was low. As expected, participants with CHD reported having greater adherence to continuous treatment than did those without the disease, and those who were using the first-line recommended drugs also reported better adherence.

Individuals with higher income reported having greater use of medication than did individuals with lower income. Moreover, access to private health insurance was associated with higher use of ARB and other antiplatelet drugs, and lower use of ACEI.

Men were more likely to use preventive drugs than women were, which may have reflected lower levels of prescription of secondary prevention for women. This highlights the importance of considering CHD among women to be a public health concern.

In Brazil, the national drug policy provides free-of-charge medication via the public healthcare system (Sistema Único de Saúde, SUS). The government has also created the Popular Pharmacy Program (Programa Farmácia Popular do Brasil), in which drugs are fully or partly financed by the government in order to increase access to medication. Initially, this program covered drugs for hypertension and diabetes. At the time of the ELSA-Brasil baseline evaluation (2008 to 2010), only two of the recommended drugs for secondary prevention of CHD were included in the Popular Pharmacy Program (beta blockers and ACEI), but all of the recommended drugs were provided via SUS.

Despite these governmental efforts to provide access, not all drugs are constantly available in the public sector, in contrast with the situation in private establishments.^27^ This could partially explain our findings of lower medication use among participants with lower income than among those with higher income. Even in the ELSA-Brasil sample, which had greater access to healthcare services and higher income than the general Brazilian population, lower mean monthly family income was still a significant barrier against use of secondary prevention.

Access to private healthcare, mainly through private health insurance, was related to lower use of standard treatment, since these people were more frequently using second-line therapies such as ARB and other antiplatelet drugs. Adverse reactions to ASA and ACEI (e.g. bleeding and coughing, respectively) are common, and could cause an increase in the use of alternative drugs (other antiplatelet medications and ARB). However, the use of these alternative drugs is considerably higher among participants with private health insurance, especially ARB use. Therefore, adverse reactions may not be the only explanation for this higher use of ARB instead of ACEI. Healthcare professionals in private institutions may be prescribing more ARB and other antiplatelet medications not only because of their possible therapeutic advantages for some CHD patients, but also because they are newer, with consequently greater pressure from the pharmaceutical industry for their prescription. Nevertheless, ASA and ACEI have been on the market for longer periods of time. They are better known and cheaper than the newer drugs, and they are still the most cost-effective option for CHD therapy.^28^^,^^29^

Frequency of medication use also differed between the sexes, such that women were less likely to use medication than men were. The women with CHD reported having lower income and education than the men in our sample. This could partially explain our findings, since these socioeconomic factors had a negative impact on medication use for secondary prevention. However, sex was associated with the use of medication independently of income and education.

Previous studies in other countries have also reported lower use of drugs among women, and have shown that they were receiving less prescription of secondary prevention for CHD than were men.^9^^,^^10^ Previous studies on ELSA-Brasil data showed that women were more aware of having hypertension and had higher frequency of medication use and blood pressure control, compared with men.^30^ They also had greater awareness about high LDL-cholesterol levels (or dyslipidemia), but had lower frequency of use of lipid-lowering medication.^31^ Thus, these studies pointed out that women may be more conscious about their health than men are, but may be receiving less prescription of medication for secondary prevention of CHD, from healthcare professionals. Atypical symptoms and underestimation of the severity of CHD among women may lead to lower or inadequate prescription of secondary prevention drugs.^9^^,^^10^ In a community-based study in Brazil, female sex presented an association with poorer one-year prognosis, and this could be explained by the lower use of secondary prevention among women.^2^ Our data allowed us to conclude that there is a group of women with low socioeconomic status that forms a risk group for CHD and for lower use of secondary prevention.

Among the risk factors for CHD, the quality of diet was better among participants who reported having previously had CHD. These individuals also reported having lower use of tobacco and alcohol, but they were less active during their leisure time, thus suggesting that it may have been easier for them to change their smoking, drinking and eating habits than to increase their frequency of physical activity practices. These individuals may feel uncomfortable or unable to practice any activity, which raises another important issue: they may not be attending cardiac rehabilitation. The frequency of attendance at cardiac rehabilitation clinics among these individuals has already been reported to be suboptimal in other studies worldwide and in Brazil.^32^^,^^33^^,^^34^

Lastly, the rates of treatment discontinuation seem to rise with increasing time elapsed since the coronary event.^3^ Our study found that participants with recent CHD had the same frequency of not using any of the recommended drugs as did those with more than 15 years since the event, but that participants with longer times since the event reported higher frequency of using only one drug. It is interesting to note that among the subjects who were using the combination recommended in the guidelines (antiplatelet plus beta blocker plus lipid-lowering drugs, with or without ACEI/ARB), the proportion of usage remained stable from the time of up to 4 years until 15 or more years since the CHD event. This suggests that this target is reachable, even if more than 15 years have elapsed since the CHD event. It is possible that the quality of medical services after a CHD event, with clear explanation about the benefits of secondary prevention, could help improve its long-term use.

This study has some strengths. ELSA-Brasil includes a large sample of men and women that enables comparisons according to sex. The sample also presents a socioeconomic gradient that enables evaluation of differences in socioeconomic levels among the participants. Moreover, this study includes detailed information about use of medications, and all the participants were instructed to bring all their medications to the study center.

Nonetheless, it also has some limitations. The previous diagnosis of CHD was self-reported, so some degree of misclassification is possible. However, other studies have shown that self-reported CHD based on information about myocardial infarction and revascularization presents good agreement with the medical records, which therefore make this useful in identifying this disease in prospective cohort studies.^35^ It is also possible that some participants did not state their medication use, even though they were asked to bring all their medications and prescriptions to the face-to-face interview, which was conducted by a well-trained interviewer using a standardized questionnaire at the ELSA-Brasil research center. In addition, it was not possible to clarify whether not using the recommended drugs was due to non-prescription by healthcare professionals or non-adherence among the participants. Moreover, although this study was based on a large and well-characterized sample, its characteristics differed from those of the general Brazilian population, especially in terms of its higher income and greater access to medical care.

## CONCLUSIONS

The use of secondary prevention of CHD was lower in this sample of middle-aged individuals than what is recommended in the standard guidelines, especially among women. These findings might be even more accentuated in the general Brazilian population, which has lower income and less access to healthcare services, compared with the ELSA-Brasil cohort. Although many strategies have been adopted over the past decades, efforts are still needed to improve the availability of effective drugs (especially in deprived areas), improve their prescription by healthcare professionals and increase patients’ awareness about the importance of adherence to secondary prevention of CHD.

## Supplemental

**Supplemental file 1.** Drugs included in categories of medication use.

Beta blockers	ACEI	ARB	Statins	Fibrates	Other antiplatelet medications
pindolol	captopril	losartan	simvastatin	bezafibrate	clopidogrel
propranolol	enalapril	valsartan	lovastatin	fenofibrate	ticlopidine
timolol	lisinopril	irbesartan	pravastatin	ciprofibrate	triflusal
sotalol	perindopril	candesartan	fluvastatin		cilostazol
nadolol	ramipril	telmisartan	atorvastatin		
metoprolol	benazepril	olmesartan	rosuvastatin		
atenolol	trandolapril				
betaxolol	delapril				
bisoprolol					
nebivolol					
carvedilol					

ACEI = angiotensin-converting enzyme inhibitor; ARB = angiotensin receptor blocker.

**Supplemental file 2.** Sociodemographic variables associated with medication use for secondary prevention of coronary heart disease, assessed using logistic regression.

	Medication use*			
	Crude		Adjusted**	
	OR	95% CI	OR	95% CI
Income				
≤ USD 1,245	Reference		Reference	
USD 1,246 to 3,319	1.154	0.654 to 2.035	1.170	0.628 to 2.180
≥ USD 3,320	2.956	1.758 to 4.970	2.537	1.082 to 5.948
Sex				
Male	Reference		Reference	
Female	0.393	0.248 to 0.623	0.520	0.316 to 0.858
Education				
Less than high school	Reference		Reference	
High school or some college/university	0.939	0.527 to 1.676	0.858	0.443 to 1.661
Completed college/university or more	1.871	1.133 to 3.091	0.724	0.338 to 1.549
Race				
White	Reference		Reference	
Mixed	0.592	0.355 to 0.989	0.960	0.533 to 1.729
Black	0.391	0.208 to 0.738	0.652	0.324 to 1.313
Asian	0.705	0.172 to 2.893	0.759	0.173 to 3.330
Indigenous	0.201	0.024 to 1.665	0.225	0.026 to 1.957
Age	1.023	0.997 to 1.050	1.004	0.974 to 1.034
Private health insurance				
No	Reference		Reference	
Yes	1.455	0.930 to 2.274	1.150	0.680 to 1.947

*Use of antiplatelet medication (acetylsalicylic acid or other) + beta blocker + lipid-lowering medication (statin or fibrate), with or without angiotensin-converting enzyme inhibitors or angiotensin-receptor blocker. **Adjusted for income, sex, education, race, age and private health insurance.

OR = odds ratio; CI = confidence interval; USD = United States dollars.

**Supplemental file 3.** Sociodemographic factors associated with sex and income of participants with coronary heart disease at ELSA-Brasil.

	Sex			Income (USD)			
	Male N = 257	Female N = 148	P-value	≤ 1,245 N = 121	1,246-3,319 N = 127	≥ 3,320 N = 154	P-value
Age (years)*	61.5 (8.2)	59.9 (7.7)	0.051	58.45 (8.0)	59.51 (8.2)	63.76 (7.1)	< 0.0001
Age stratum (%)			0.420				< 0.0001
35-44	3 (1.2)	4 (2.7)		5 (4.1)	2 (1.6)	0 (0.0)	
45-54	53 (20.6)	33 (22.3)		33 (27.3)	37 (29.1)	16 (10.4)	
55-64	106 (41.2)	66 (44.6)		57 (27.3)	49 (38.6)	66 (42.9)	
65-74	95 (37.0)	45 (30.4)		26 (21.5)	39 (30.7)	72 (46.8)	
Female (%)	-	-	-	64 (52.9)	58 (45.7)	28 (16.2)	< 0.0001
Race (%)			< 0.0001				< 0.0001
White	155 (62.0)	57 (39.0)		38 (31.7)	48 (38.4)	126 (83.4)	
Mixed	53 (21.2)	45 (30.8)		44 (36.7)	44 (35.2)	10 (6.6)	
Black	30 (12.0)	39 (26.7)		34 (28.3)	27 (21.6)	8 (5.3)	
Asian	6 (2.4)	3 (2.1)		1 (0.8)	4 (3.2)	4 (2.6)	
Indigenous	6 (2.4)	2 (1.4)		3 (2.5)	2 (1.6)	3 (0.8)	
Educational level (%)			< 0.0001				< 0.0001
Less than high-school	64 (24.9)	50 (33.8)		70 (57.9)	36 (28.3)	7 (4.5)	
High school and some college/university	44 (17.1)	50 (33.8)		42 (34.7)	48 (37.8)	4 (4.3)	
Completed college/university or more	149 (58.0)	48 (32.4)		9 (7.4)	43 (33.9)	143 (92.9)	
Mean monthly family income (%)			< 0.0001				
≤ USD 1,245	57 (22.4)	64 (43.5)		-	-	-	-
USD 1,246-3,319	69 (27.1)	58 (39.5)		-	-	-	-
≥ USD 3,320	129 (50.6)	25 (17.0)		-	-	-	-
Private health insurance (%)	178 (69.3)	94 (63.5)	0.236	56 (46.3)	83 (65.4)	130 (84.4)	< 0.0001

*Presented as mean (standard deviation). USD = United States dollars.

## References

[1] GBD 2016 Causes of Death Collaborators (2017). Global, regional, and national age-sex specific mortality for 264 causes of death, 1980-2016: a systematic analysis for the Global Burden of Disease Study 2016. Lancet.

[2] Santos IS, Goulart AC, Brandão RM (2015). One-year mortality after an acute coronary event and its clinical predictors: The ERICO study. Arq Bras Cardiol.

[3] Ergatoudes C, Thunstrom E, Rosengren A (2016). Long-term secondary prevention of acute myocardial infarction (SEPAT) - guidelines adherence and outcome. BMC Cardiovasc Disord.

[4] Piegas LS, Timerman A, Nicolau JC (2004). III Diretriz sobre tratamento do infarto agudo do miocárdio [III Guidelines on the treatment of myocardial acute infarction]. Arq Bras Cardiol.

[5] Lima RC, Kubrusly LF, Nery ACS (2004). Sociedade Brasileira de Cardiologia. Diretrizes da Cirúrgia de Revascularização Miocárdica [Guidelines for myocardial revascularization surgery].. Arq Bras Cardiol.

[6] Smith SC, Allen J, Blair SN, AHA/ACC; National Heart, Lung, and Blood Institute (2006). AHA/ACC guidelines for secondary prevention for patients with coronary and other atherosclerotic vascular disease: 2006 update: endorsed by the National Heart, Lung, and Blood Institute. Circulation.

[7] O’Gara PT, Kushner FG, Ascheim DD (2013). 2013 ACCF/AHA guideline for the management of ST-elevation myocardial infarction: a report of the American College of Cardiology Foundation/American Heart Association Task Force on Practice Guidelines. Circulation.

[8] Levine GN, Bates ER, Blankenship JC (2011). 2011 ACCF/AHA/SCAI guideline for percutaneous coronary intervention: a report of the American College of Cardiology Foundation/American Heart Association Task Force on Practice Guidelines and the Society for Cardiovascular Angiography and Interventions. Circulation.

[9] Koopman C, Vaartjes I, Heintjes EM (2013). Persisting gender differences and attenuating age differences in cardiovascular drug use for prevention and treatment of coronary heart disease, 1998-2010. Eur Heart J.

[10] Manteuffel M, Williams S, Chen W (2014). Influence of patient sex and gender on medication use, adherence, and prescribing alignment with guidelines. J Womens Health (Larchmt).

[11] Du L, Cheng Z, Zhang Y, Li Y, Mei D (2017). The impact of medication adherence on clinical outcomes of coronary artery disease: A meta-analysis. Eur J Prev Cardiol.

[12] Reuter H, Markhof A, Scholz S (2015). Long-term medication adherence in patients with ST-elevation myocardial infarction and primary percutaneous coronary intervention. Eur J Prev Cardiol.

[13] Aquino EM, Barreto SM, Benseñor IM (2012). Brazilian Longitudinal Study of Adult Health (ELSA-Brasil): Objectives and Design. Am J Epidemiol.

[14] Schmidt MI, Duncan BB, Mill JG (2015). Cohort Profile: Longitudinal Study of Adult Health (ELSA-Brasil). Int J Epidemiol.

[15] Bensenor IM, Griep RH, Pinto KA (2013). Routines of organization of clinical tests and interviews in the ELSA-Brasil investigation center. Rev Saude Publica.

[16] Chor D, Alves MG, Giatti L (2013). Questionário do ELSA-Brasil: desafios na elaboração de instrumento multidimensional [Questionnaire development in ELSA-Brasil: challenges of a multidimensional instrument]. Rev Saúde Pública.

[17] Aquino EM, Vasconcellos-Silva PR, Coeli CM (2013). Aspectos éticos em estudos longitudinais: o caso do ELSA-Brasil [Ethical issues in longitudinal studies: the case of ELSA-Brasil]. Rev Saúde Pública.

[18] Lohman TG, Roche AF, Martorell R (1988). Anthropometric standardization reference manual.

[19] Fedeli LG, Vidigal PG, Leite CM (2013). Logistics of collection and transportation of biological samples and the organization of the central laboratory in the ELSA-Brasil. Rev Saude Publica.

[20] Lewis G, Pelosi AJ, Araya R, Dunn G (1992). Measuring psychiatric disorder in the community: a standardized assessment for use by lay interviewers. Psychol Med.

[21] Nunes MA, de Mello Alves MG, Chor D, Schmidt MI, Duncan BB (2012). Cross-cultural adaptation of CIS-R (Clinical Interview Schedule-Revised Version) for the Portuguese in Longitudinal Study Of Adult Health (ELSA) [Adaptação transcultural do CIS-R (Clinical Interview Schedule - Revised Version) para o português no Estudo Longitudinal de Saúde do Adulto (ELSA)]. Revista HCPA.

[22] Previdelli AN, Andrade SC, Pires MM (2011). A revised version of the Healthy Eating Index for the Brazilian population. Rev Saude Publica.

[23] Craig CL, Marshall AL, Sjöström M (2003). International physical activity questionnaire: 12-country reliability and validity. Med Sci Sports Exerc.

[24] Matsudo S, Araújo T, Matsudo V (2001). International physical activity questionnaire (IPAQ): study of validity and reliability in Brazil [Questionário internacional de atividade física (IPAQ): Estudo de validade e reprodutibilidade no Brasil]. Rev bras ativ fís saúde.

[25] Treff C, Benseñor IM, Lotufo PA (2017). Leisure-time and commuting physical activity and high blood pressure: the Brazilian Longitudinal Study of Adult Health (ELSA-Brasil). J Hum Hypertens.

[26] Morisky DE, Green LW, Levine DM (1986). Concurrent and predictive validity of a self-reported measure of medication adherence. Med Care.

[27] Dal Pizzol TS, Trevisol DJ, Heineck I (2010). Adherence to essential medicines in cities from three Brazilian states. Cad Saúde Pública.

[28] Jackevicius CA, Cox JL, Carreon D (2009). Long-term trends in use of and expenditures for cardiovascular medications in Canada. CMAJ.

[29] Sheppard RJ, Schiffrin EL (2013). Inhibition of the renin-angiotensin system for lowering coronary artery disease risk. Curr Opin Pharmacol.

[30] Chor D, Pinho Ribeiro AL, Sá Carvalho M (2015). Prevalence, Awareness, Treatment and Influence of Socioeconomic Variables on Control of High Blood Pressure: Results of the ELSA-Brasil Study. PLoS One.

[31] Lotufo PA, Santos RD, Figueiredo RM (2016). Prevalence, awareness, treatment, and control of high low-density lipoprotein cholesterol in Brazil: Baseline of the Brazilian Longitudinal Study of Adult Health (ELSA-Brasil). J Clin Lipidol.

[32] Kweon S, Sohn MK, Jeong JO (2017). Quality of Life and Awareness of Cardiac Rehabilitation Program in People With Cardiovascular Diseases. Ann Rehabil Med.

[33] Ghisi GL, dos Santos RZ, Aranha EE (2013). Perceptions of barriers to cardiac rehabilitation use in Brazil. Vasc Health Risk Manag.

[34] Borghi-Silva A, Mendes RG, Trimer R, Cipriano G (2014). Current trends in reducing cardiovascular disease risk factors from around the world: focus on cardiac rehabilitation in Brazil. Prog Cardiovasc Dis.

[35] Okura Y, Urban LH, Mahoney DW, Jacobsen SJ, Rodeheffer RJ (2004). Agreement between self-report questionnaires and medical record data was substantial for diabetes, hypertension, myocardial infarction and stroke but not for heart failure. J Clin Epidemiol.

